# Adaptation of left ventricular diastolic function to pregnancy: a systematic review and meta-analysis

**DOI:** 10.1097/HJH.0000000000002886

**Published:** 2021-05-17

**Authors:** Sander de Haas, Marc E.A. Spaanderman, Sander M.J. van Kuijk, Joris van Drongelen, Zenab Mohseni, Laura Jorissen, Chahinda Ghossein-Doha

**Affiliations:** a Department of Obstetrics and Gynaecology; b Department of Clinical Epidemiology and Medical Technology Assessment, Maastricht University Medical Center (MUMC+); c Department of Obstetrics and Gynaecology, Radboud University Nijmegen Medical Center; d Department of Cardiology, Maastricht University Medical Center (MUMC+), The Netherlands

**Keywords:** hypertensive pregnancy, left ventricular diastolic dysfunction, left ventricular diastolic function, physiology, preeclampsia, pregnancy

## Abstract

**Objective:**

To meta-analytically determine the adaptation of left ventricular diastolic function (LVDF)-indices to singleton normotensive pregnancies.

**Methods:**

Literature was retrieved from PubMed and Embase. We included studies that reported a nonpregnant reference measurement and LVDF indices (mitral inflow signals, left atrial volume and tissue Doppler measurements). Mean differences between pregnant and reference measurements and weighted means of absolute values were calculated using a random-effects model.

**Results:**

We included 34 eligible studies. Normotensive pregnancies were characterized by an initially larger increase in the passive left ventricular filling (E-wave peak velocity, 13%) compared to active left ventricular filling during diastole (A-wave peak velocity, 6%) resulting in a 16% increase of the E/A ratio in the first trimester. The E/A ratio progressively decreased during advancing gestation to −18% at term, resulting from stabilizing E-wave peak velocity and increased A-wave peak velocity. The E/e′ ratio was increased between 22 and 35 weeks (a maximal increase of 13%) in normotensive pregnancy. Left atrial volume (LAV) progressively increased from 15 weeks onwards with a maximal increase of 30% between 36 and 41 weeks.

**Conclusion:**

LVDF in normotensive pregnancy was improved in the first trimester after which LVDF progressively worsened. Large-scale studies in normotensive and hypertensive complicated pregnancies are needed for a more precise insight into LVDF changes during pregnancy.

## INTRODUCTION

During pregnancy, the maternal cardiovascular system undergoes significant adaptive changes, including a slightly decreased blood pressure, increased cardiac output and plasma volume expansion [1,2]. These changes are initiated by a profound reduction in cardiac afterload due to systemic arterial dilation [1–3]. On the one hand, pregnancy-induced plasma volume expansion is paralleled by increased ventricular dimensions, on the other hand with augmented left ventricular (LV) mass [4]. In order to withstand elevated resting cardiac output without a sustained increased sympathetic-tone reliant increase in heart rate and contractility, cardiac compliance and with it left ventricular diastolic function (LVDF) should improve to allow increased filling. A healthy pregnancy-associated eccentric hypertrophy is likely to improve LVDF [5]. In contrast, the much more pronounced concentric hypertrophy of hypertensive complicated pregnancies is strongly related to impaired LVDF [4,6,7]. This type of hypertrophy often persists subclinically after pregnancy and may predispose to the increased risk for remote cardiovascular morbidity and mortality in these women later on [8,9]. Timely detection of aberrant gestational adjustments may allow timely instituted interventions to promote healthy cardiac remodeling before clinical problems arise. Although it is currently known that LVDF changes during pregnancy, studies have not been able to consistently report the magnitude and time-course of change [7,10]. Inconsistencies may relate to different cardiac measurements used, heterogeneity in the studied population, study design and the used comparison groups. Therefore, we performed a systematic review with meta-analysis of the current literature to estimate the extent and time course of changes in LVDF during singleton normotensive pregnancies.

## METHODS

### Literature search

An extensive systematic literature search was conducted on articles evaluating LVDF during normotensive pregnancies using the PubMed (NCBI) and Embase (Ovid) databases. PubMed and Embase provided publications ranging from 1946 and 1974 to July 2020, respectively. The search strategy was originally designed for a series of meta-analyses on cardiac geometry, left ventricular systolic function, and LVDF with a focus on normotensive and hypertensive pregnancies, as detailed in Table 1, Supplemental Digital Content.

### Selection of studies

**FIGURE 1:**
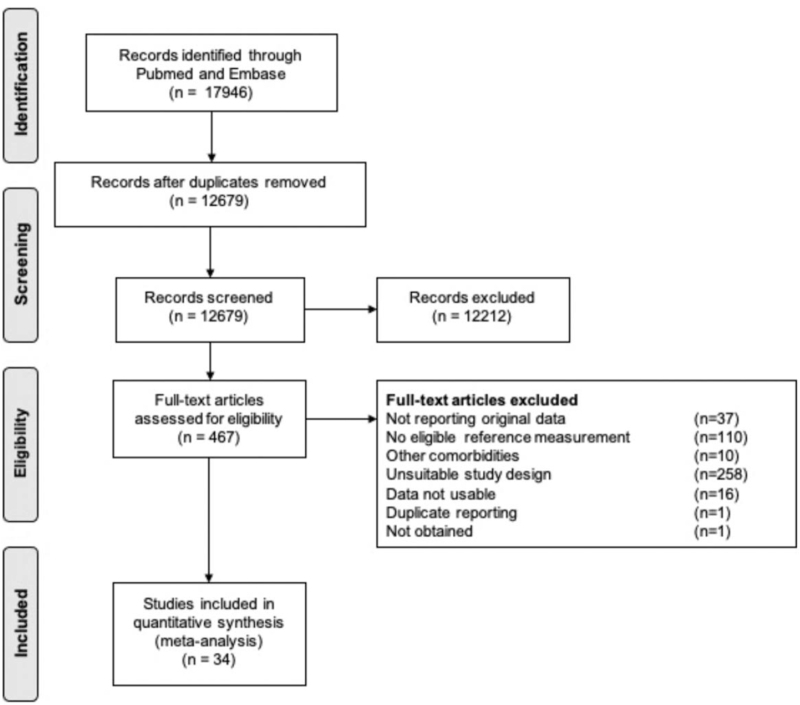
Flowchart summarizing the process of study selection.

The identified articles were assessed for eligibility in two phases (Fig. 1). First, all studies were independently screened for eligibility based on the title and abstract by two authors (S.dH. and C.G.D. or L.J.). Second, full-text articles were screened independently for eligibility based on the inclusion and exclusion criteria by the same authors. Discrepancies were resolved by mutual agreement.

Studies were included if they reported a reference measurement, either estimated before conception, at or after 6 weeks postpartum, or in nonpregnant controls, and at least one measurement during pregnancy at any gestational age. Studies needed to report numerical values (mean) with standard deviation (SD), standard error (SE), or a 95% confidence interval (95% CI)). Data was requested in the correct format from authors if they reported their data differently. Exclusion criteria included studies with a study population consisting of women with preexisting cardiovascular disease, or with a history of a hypertensive pregnancy in a previous pregnancy, articles in other languages than English or Dutch, and case reports.

### Data extraction

Study characteristics (study design, sample size of study population and methods used), anthropometric measures (age, nonpregnant weight, height, parity, and gestational age at measurements), and effective measures with SD, SE, or 95% CI were collected from the eligible studies in predesigned data collection forms. LVDF indices collected and detailed were mitral E-wave peak velocity (E-peak), mitral A-wave peak velocity (A-peak), E/A ratio, left atrial volume (LAV), and E/e′ ratio.

### Quality assessment

The eligible studies were all assessed for study quality and risk of bias using a modified list of items described in the Quality In Prognosis Studies (QUIPS) tool, which was adapted for the purposes of this review [11]. Studies were scored with a plus (+) or minus (−) for risk of bias on five domains including study participation, study attrition, variable measurement, data reporting, and study design. Items that were not applicable for a study were assigned with question marks. Studies were only scored for study attrition if the study conducted a longitudinal prospective study. Studies with a positive score >60% were identified as high quality (HQ) studies, whereas studies with a score lower than 30% were identified as low quality (LQ) studies. Studies were identified as moderate quality (MQ) studies if the study scored between 30% and 60%.

### Statistical analysis

The gestational age was categorized in five different intervals of gestational age (<14, 15–21, 22–28, 29–35, and 36–41 weeks of pregnancy). These gestational age intervals were adapted from Abudu *et al.* and enabled the best categorization of all indices based on the categorization of available literature [12]. If necessary, the SD was calculated from SE or 95% CI according to the Cochrane handbook for systematic reviews of interventions [13]. The changes of the different indices compared to the reference measurements and pooled absolute values were calculated separately for the predefined gestational age intervals using a random-effects model as earlier described by DerSimonian and Laird [14]. The random-effects model allows for inter-study variation and was chosen because observational data of different pregnant populations were used in terms of anthropometric and clinical characteristics. Egger's regression test for funnel plot asymmetry was conducted to test for publication bias in each gestational age interval [15]. Primary outcomes were corrected for publication bias using the Trim and Fill method, as described by Duval and Tweedie [16]. The primary outcomes for each study were the mean difference (MD) of the indices for LVDF between pregnancy and the reference group which is also presented as relative increase from a reference in percentage. A pooled estimate of the absolute values for the LVDF indices are also reported. The outcomes are reported with a 95% CI. The ratio between total heterogeneity and total variability (*I*-squared statistic (*I*^2^)) is presented as a measure for heterogeneity. *I*^2^ can distinguish true heterogeneity from sampling variance and is expressed as a percentage [17]. Sources of heterogeneity (reference type and quality of study) were investigated by meta-regression analyses using a mixed-effects model. The meta-analyses were performed in R version 4.0.2 using the meta package version 4.13-0

## RESULTS

### Study and data selection

The search strategy resulted in 12 679 unique articles. Articles published in languages other than Dutch or English were manually excluded (*n* = 1605). Finally, 34 studies were eligible for inclusion (Fig. 1) [5,10,18–49]. We converted the data of one study [5] according to the recommendations by Cochrane. We suspected double reporting of one article [18,50], and one article was not possible to retrieve [51].

All studies reported data on normotensive pregnancies. Because of insufficient individual data on E/e′ ratios calculated with lateral e′ and septal e′, we used the E/e′ ratios from studies using the average e′. Two studies did not report if they used the lateral, septal or average e′. We treated the ratio as being the average e′ [19,20].

### Study characteristics and quality assessment

The study characteristics for the included studies are depicted in Table 2, Supplemental Digital Content. All studies used transthoracic echocardiography (TTE) and Doppler or tissue Doppler imaging (TDI). The results of the quality assessment are depicted in Table 3, Supplemental Digital Content.

### E-peak

**FIGURE 2:**
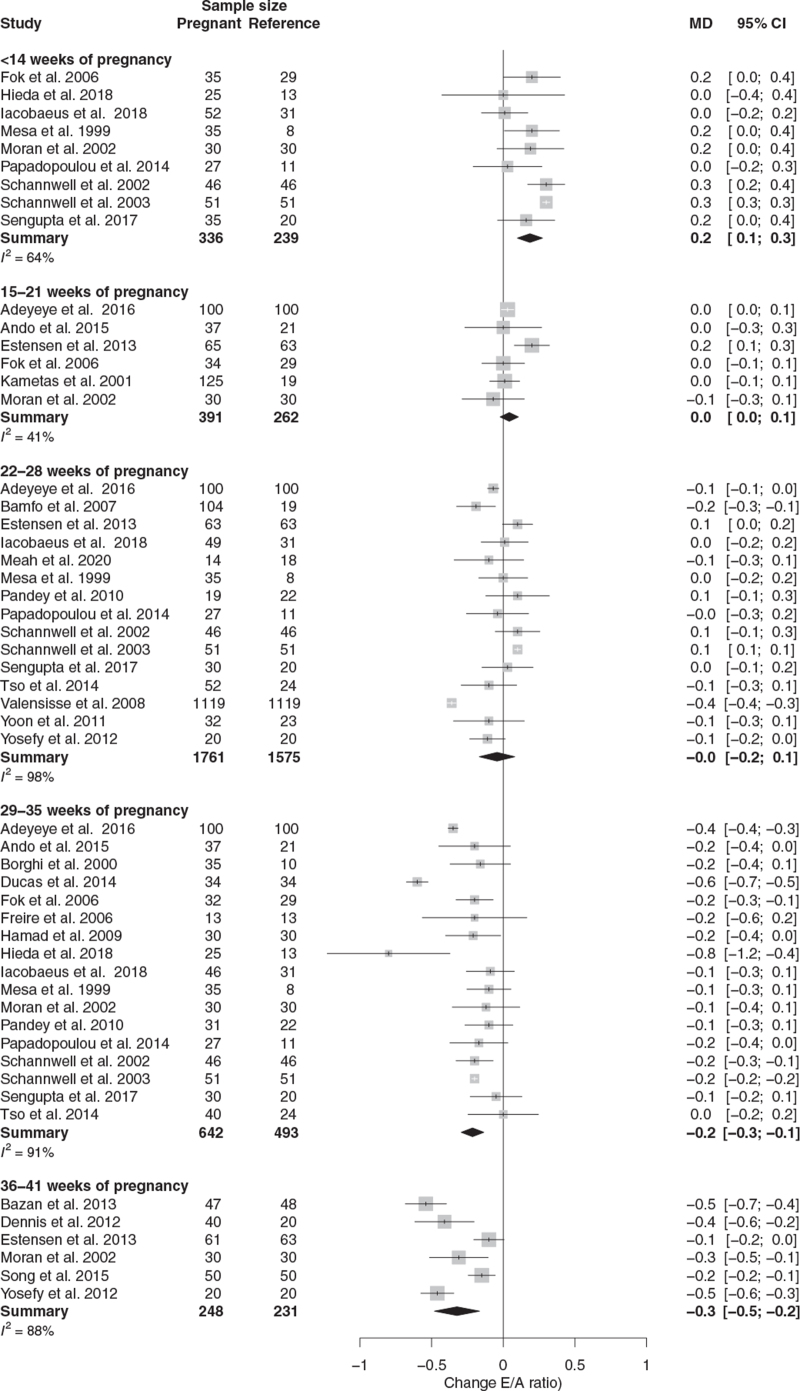
Forest plot of E/A ratio change during normotensive pregnancy.

In normotensive pregnancies, E-peak increased in the first 21 weeks of pregnancy (Table 1, Fig. 1, Supplemental Digital Content). From 22 weeks of pregnancy onwards the E-peak normalized to reference values. Absolute values of the E-peak progressively decreased from the first trimester onwards (Table 1, Fig. 2, Supplemental Digital Content).

**TABLE 1:** Pooled changes of left ventricular diastolic function indices in normotensive pregnancies

	Gestational age interval (weeks)					
	Reference	<14	15–21	22–28	29–35	36–41
E-peak (m s^−1^)						
Abs	0.83 (0.79 to 0.87)	0.89 (0.87 to 0.91)	0.88 (0.81 to 0.96)	0.87 (0.84 to 0.90)	0.83 (0.80 to 0.87)	0.80 (0.74 to 0.87)
MD	–	0.10 (0.07 to 0.12)	0.14 (0.11 to 0.18)	0.04 (−0.02 to 0.11)	0.02 (−0.02 to 0.07)	−0.02 (−0.04 to 0.01)
%	–	12.5 (9.4 to 5.7)	19.2 (14.4 to 24.0)	5.1 (−2.6 to 12.9)	3.0 (−3.0 to 9.0)	−1.9 (−5.0 to 1.3)
cMD	–	–	–	–	0.11 (0.07 to 0.16)	–
A-peak (m s^−1^)						
Abs	0.52 (0.49 to 0.56)	0.55 (0.52 to 0.57)	0.57 (0.50 to 0.63)	0.60 (0.54 to 0.65)	0.65 (0.58 to 0.72)	0.59 (0.53 to 0.65)
MD	–	0.03 (0.00 to 0.06)	0.08 (0.04 to 0.12)	0.07 (0.04 to 0.10)	0.13 (0.06 to 0.19)	0.10 (0.04 to 0.17)
%	–	6.1 (0.5 to 11.8)	16.0 (7.5 to 24.5)	12.8 (6.7 to 18.8)	24.2 (11.4 to 36.9)	21.0 (7.3 to 34.6)
cMD	–	–	–	–	0.21 (0.15 to 0.28)	–
E/A ratio						
Abs	1.6 (1.5 to 1.7)	1.7 (1.4 to 1.9)	1.6 (1.5 to 1.7)	1.5 (1.4 to 1.6)	1.4 (1.3 to 1.4)	1.4 (1.3 to 1.5)
MD	–	0.2 (0.1 to 0.3)	0.0 (0.0 to 0.1)	−0.0 (−0.2 to 0.1)	−0.2 (−0.3 to −0.1)	−0.3 (−0.5 to −0.2)
%	–	15.9 (11.2 to 20.5)	2.7 (−1.4 to 6.8)	−3.0 (−11.2 to 5.2)	−14.4 (−19.6 to −9.1)	−18.4 (−27.4 to −9.5)
cMD	–	0.3 (0.2 to 0.4)	–	–	–	–
LAV (ml)						
Abs	36 (29 to 42)	41 (14 to 68)	46 (43 to 49)	45 (21 to 70)	49 (41 to 56)	46 (28 to 65)
MD	–	4 (−2 to 11)	7 (3 to 11)	9 (5 to 13)	11 (9 to 12)	10 (8 to 13)
%	–	12.0 (−6.7 to 30.8)	19.5 (9.0 to 30.0)	24.9 (13.8 to 35.9)	28.9 (23.7 to 34.1)	29.6 (22.1 to 37.1)
cMD	–	–	–	–	–	–
E/e′ ratio						
Abs	6.6 (5.9 to 7.4)	6.6 (6.0 to 7.1)	6.1 (5.5 to 6.7)	6.8 (6.3 to 7.3)	7.6 (6.2 to 9.0)	6.6 (5.8 to 7.4)
MD	–	0.4 (0.0 to 0.8)	0.3 (−0.1 to 0.7)	0.6 (0.2 to 0.9]	0.8 (0.4 to 1.3)	0.0 (−0.9 to 0.9)
%	–	6.8 (0.3 to 13.3)	5.8 (−1.4 to 12.9)	9.2 (4.0 to 14.3)	12.8 (5.5 to 20.1)	0.0 (−13.2 to 13.2)
cMD	–	–	–	–	–	–

Values are reported as mean difference (MD) and relative change (%) with 95% confidence interval (CI) compared to the reference group and absolute values (Abs) with 95% CI. MD corrected for publication bias (cMD) are also presented for intervals with statistically significant funnel plot asymmetry.

LAV, left atrial volume.

Publication bias was present in the 29 to 35 weeks interval (*P* *<* 0.01). The corrected MD is presented in Table 1. Neither type of reference (PP vs. NP and PC, *P* *=* 0.67; PC vs. PP and NP *P* = 0.23*)* nor study quality (LQ vs. MQ and HQ, *P* = 0.20 and MQ vs. LQ and HQ, *P* = 0.60) contributed to the observed heterogeneity.

### A-peak

**FIGURE 3:**
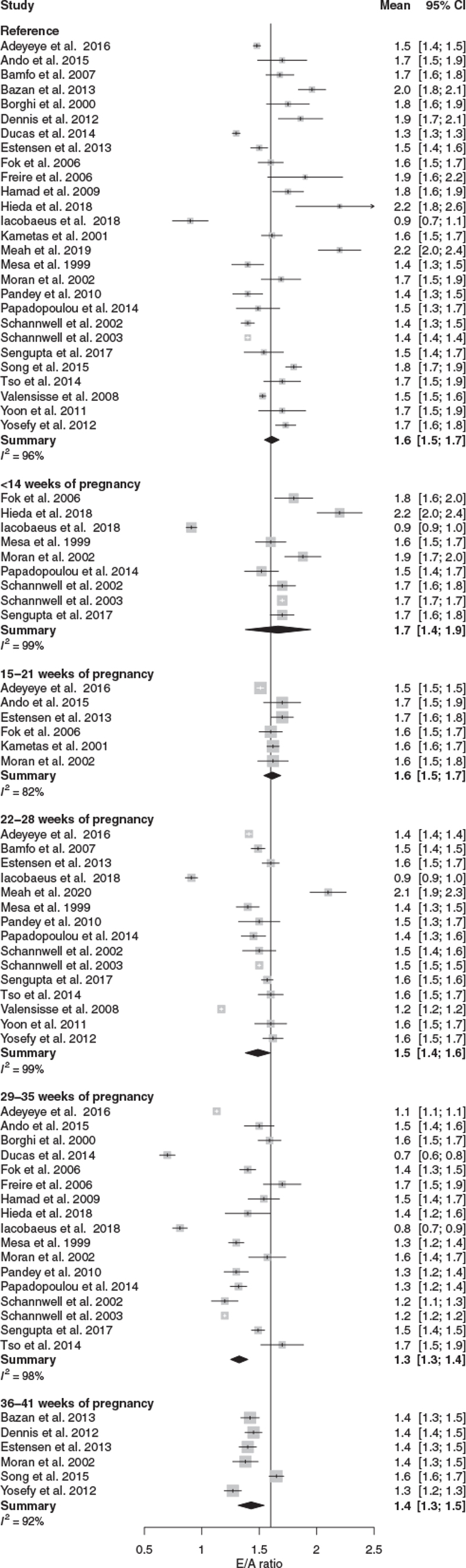
Forest plot of absolute E/A ratio values during normotensive pregnancy.

In normotensive pregnancy, A-peak progressively increased from 15 weeks onwards with a maximal increase between 29 and 35 weeks (Table 1, Figs. 3 and 4, Supplemental Digital Content).

Publication bias was present in the 29–35 weeks interval (*P* = 0.005). The corrected MD is presented in Table 1. Neither type of reference (PP vs. NP and PC, *P* = 0.52; PC vs. PP and NP, *P* = 0.81) nor study quality affected the observed adjustments (LQ vs. MQ and HQ, *P* = 0.55; MQ vs. LQ and HQ, *P* = 0.96).

### E/A ratio

The E/A ratio increased in the first 14 weeks and progressively decreased from the early second trimester onwards reaching a significant decrease between 29 and 35 weeks (Table 1, Figs. 2 and 3).

Publication bias was present in the <14 weeks of pregnancy interval (*P* = 0.008). The corrected MD is presented in Table 1. Type of reference contributed to the observed heterogeneity (PP vs. NP and PC, *P* *=* 0.37; PC vs. PP and NP *P* = 0.03). Study quality did not statistically significant contribution to the observed heterogeneity (LQ vs. MQ and HQ, *P* = 0.46; MQ vs. LQ and HQ, *P* = 0.87)).

### Left atrial volume

In normotensive pregnancies, LAV progressively increased from 15 weeks onwards with a maximal increase between 36 and 41 weeks (Table 1, Figs. 5 and 6, Supplemental Digital Content).

Publication bias could not be tested in all intervals because of insufficient studies, except for the 29 to 35 weeks interval (*P* = 0.30).

All studies that reported data on LAV during pregnancy were scored as MQ studies. Type of reference was not associated with the LAV change (*P* = 0.27).

### E/e′ ratio

In normotensive pregnancies, the E/e′ ratio changed only between 22 and 35 weeks (Table 1, Figs. 7 and 8, Supplemental Digital Content).

Publication bias could only be estimated in the <14, 15–21, 22–28, and 29–35 weeks intervals (*P* = 0.91, *P* = 0.94, *P* = 0.60 and *P* = 0.40), respectively. No contributors to the heterogeneity were observed (PC vs. PP and NP, *P* = 0.24; PP vs. NP and PC, *P* *=* 0.47; and MQ vs. HQ, *P* = 0.20).

## DISCUSSION

This systematic review with meta-analysis evaluates the adaptation of LVDF in normotensive pregnancies. Diastolic function is the relaxation phase during the cardiac cycle when the ventricles fill with blood after systole. To the best of our knowledge, this is the first meta-analysis evaluating the adaptation of LVDF to pregnancy.

Normotensive pregnancy is characterized by a continuous state of increased cardiac preload, initially due to venoconstriction and later on due to increasing circulating volume [3,4]. In nonpregnant conditions, up to 80% of the ventricular filling occurs via early passive filling (E-peak) [21]. In the first trimester of pregnancy, the E-peak increases more than the A-peak, resulting in an increased E/A ratio of 16%. This suggests a state of above average early passive filling caused by an increased pressure gradient between the LA and LV. The increased pressure gradient is reached by a gain in extra circulatory volume along with reduced LA afterload, increased respiration and venoconstriction [4,5,22,52]. From the second trimester onwards, the E/A ratio steadily decreases with a maximal decrease of 18% at term. The decline of the E/A ratio is built upon a progressive increase of the A-peak up to 24%. The E-peak is only increased up to 21 weeks of pregnancy after which it gradually decreases towards reference at term. As a single variable, the decrease in E/A ratio does not necessarily reflect a gradual impairment of LVDF but might suggest a trend towards impaired LV relaxation throughout pregnancy. The LV adapts to the continuous strain of increasing volume load in advancing gestation with LV hypertrophy [4,5]. This is likely to decrease LV compliance, eventually resulting in higher LV end-diastolic pressures [52]. According to the Frank-Starling mechanism, increased preload results in increased cardiac output but also to a shift in the pressure-volume curve to the right, eventually resulting in reduced LV compliance due to excessive stretch of the cardiac myocytes [53]. The hypothesis that LV compliance is diminished is supported by the up to 13% increase of the E/e′ ratio.

To maintain adequate LV filling, the LA compensates for the increased end-diastolic pressures with an increased atrial kick, as reflected by the increased A-peak. The increased venous return to the LA and the decreased LV compliance is also reflected by the progressive increase of the LAV. LA remodeling is known to play an important role in LVDF and therefore has an indirect effect on overall cardiac performance [21,23]. If LA emptying is impaired, the atrial pressure increases to optimize the pressure gradient, thereby maintaining proper LV filling. Eventually, impaired emptying results in LA overstretching and dilatation to accommodate the increased preload, as is obviously the case in pregnancy [21,23]. Increased plasma atrial natriuretic protein concentrations in pregnancy support this hypothesis [54–56].

Unfortunately, longitudinal studies investigating cardiac function in hypertensive pregnancies were very scarce and measurements before 22 weeks of pregnancy were not made, making it impossible to evaluate early adaption of LVDF in women destined to develop gestational hypertensive disorders.

### Clinical implications and recommendations for future research

The adaptation of LVDF might give additional insights on the cardiovascular adaptation to the hemodynamic load in individual pregnancies in combination with other hemodynamic parameters. Due to the lack of data on LVDF in hypertensive pregnancies, we do not believe that routinely evaluation of LVDF has the potential to discriminate between hemodynamic maladaptive pregnancies and healthy pregnancies. This is relevant because decreased LVDF might precede impaired systolic function and therefore might be helpful to identify high-risk pregnancies.

LVDF indices are traditionally measured by TTE in combination with Doppler flow measurements. These traditional measurements are well defined and have been widely applied in echocardiographic studies. A disadvantage is that these measurements are dependent on cardiac loading conditions [57]. TDI is less load-dependent, suggesting that TDI is more appropriate [24]. Another promising and less load-dependent and angle-independent measurement modality is speckle tracking echocardiography (STE), which is capable to assess more subtle functional myocardial abnormalities [58].

With this meta-analysis we intend to change future study designs that aim at assessing cardiac function during pregnancy, as follows: more traditional doppler parameters should be combined with the less load-dependent TDI and ideally with STE; relevant hormonal, metabolic, geometrical and hemodynamic parameters should be clearly described, along with circulation-modulating drugs used by patients with hypertensive pregnancies; large cohort studies should be set up to allow first and early second trimester changes in cardiac function; different subtypes of gestational hypertensive pregnancies should be evaluated separately.

### Limitations

Some longitudinal studies used a postpartum reference and reported pregnant data of the same women. These studies did not report sufficient information to determine the covariance between both measurements. Instead of estimating covariances, their dependence was ignored. We expect that this decision could only have resulted in slightly conservative estimates of precision. Second, the power was not always high enough to reliably test for publication bias. Therefore, the corrected MD should be interpreted with caution.

The observed heterogeneity could not always be explained by the study quality and reference group used. It is very likely that other clinically relevant factors are involved, including parity and anthropometric measures. These factors were insufficiently reported, making it impossible to explore their combined relationship.

In conclusion, the change in LVDF in early normotensive pregnancy was characterized by an increased contribution of passive LV filling, which resulted in an increased E/A ratio. In advancing gestation, the change in LVDF was characterized by increased active LV filling, which resulted in a progressive decrease of the E/A ratio in the second half of pregnancy. Large-scale studies in normotensive and hypertensive complicated pregnancies are needed for a more precise insight into LVDF changes during pregnancy.

## ACKNOWLEDGEMENTS

Funding: None.

Disclosures: None.

### Conflicts of interest

There are no conflicts of interest.

## Supplementary Material

Supplemental Digital Content
