# Supplementary material for: Adaptation of left ventricular diastolic function to pregnancy: a systematic review and meta-analysis
Source: J Hypertens. 2021 May 17;39(10):1934–41. doi: 10.1097/HJH.0000000000002886 (PMC8452327; doi:10.1097/HJH.0000000000002886)
Supplement: Supplemental Digital Content [file jhype-39-1934-s001.docx]

**SUPPLEMENTAL MATERIAL**

**Adaptation of left ventricular diastolic function to pregnancy: a systematic review and meta-analysis**

Sander de Haas^A^, MD

Marc EA Spaanderman^A^, MD PhD

Sander MJ van Kuijk^B^, PhD

Joris van Drongelen^C^, MD PhD

Zenab Mohseni^A^, BSc

Laura Jorissen^A^, MD

Chahinda Ghossein-Doha^D^, MD PhD

| ^A^ | *Department of Obstetrics and Gynaecology, Maastricht University Medical Center (MUMC+), The Netherlands* |
| --- | --- |
| ^B^ | *Department of Clinical Epidemiology and Medical Technology Assessment, Maastricht University Medical Center (MUMC+), The Netherlands.* |
| ^C^ | *Department of Obstetrics and Gynaecology, Radboud University Nijmegen Medical Center, The Netherlands* |
| ^D^ | *Department of Cardiology, Maastricht University Medical Center (MUMC+), The Netherlands* |

**Supplemental Table 1.** Literature search strategy for PubMed (MEDLINE) and Embase (Ovid)

| **Component** | **PubMed (MEDLINE)** | **Embase (Ovid)** |
| --- | --- | --- |
| 1. Pregnancy | pregnancy [Mesh] OR pregnancy [tiab] OR pregnancies [tiab] OR pregnant [tiab] OR gestation [tiab] OR gestations [tiab] OR gestational [tiab] OR gravidity [Mesh] OR gravidity [tiab] OR gravidities [tiab] OR gravid [tiab] | exp pregnancy/ or exp gravidity/ or exp gestation/ or (pregnancy or pregnancies or pregnant or gestation or gestations or gestational or gravidity or gravidities or gravid).ti,ab. |
| 1. PIH | "hypertension, pregnancy-induced" [Mesh] OR "pregnancy induced hypertension" [tiab] OR "pregnancy associated hypertension" [tiab] OR PIH [tiab] OR “hypertensive pregnancy” [tiab] OR “pregnancy hypertension” [tiab] OR “gestational hypertension” [tiab] OR “HELLP syndrome” [Mesh] OR “HELLP” [tiab] OR “Hemolysis, Elevated Liver Enzymes, Lowered Platelets” [tiab] | exp maternal hypertension/ or exp HELLP syndrome/ or (pregnancy induced hypertension or pregnancy associated hypertension or PIH or hypertensive pregnancy or pregnancy hypertension or gestational hypertension or HELLP or Hemolysis, Elevated Liver Enzymes, Lowered Platelets).ti,ab. |
| 1. FGR | "fetal growth retardation" [tiab] OR "fetal growth retardation" [tiab] OR “fetal growth restriction” [tiab] OR FGR [tiab] OR "intrauterine growth retardation" [tiab] OR “intrauterine growth restriction” [tiab] OR IUGR [tiab] OR "Infant, Small for Gestational Age" [Mesh] OR “small for gestational age” [tiab] OR SGA [tiab] | exp intrauterine growth retardation/ or exp small for date infant/ or (fetal growth retardation or fetal growth restriction or FGR or intrauterine growth retardation or intrauterine growth restriction or IUGR or small for gestational age or SGA).ti,ab. |
| 1. PE | "pre-eclampsia" [Mesh] OR "pre-eclampsia" [tiab] OR preeclampsia [tiab] OR preeclamptic [tiab] OR pre-eclamptic [tiab] or PE [tiab] OR “eclampsia” [Mesh] OR eclampsia [tiab] OR eclampsias [tiab] OR eclamptic [tiab] OR toxemia [tiab] OR toxemias [tiab] | exp preeclampsia/ or exp eclampsia/ or (pre-eclampsia or preeclampsia or pre-eclamptic or preeclamptic or PE or eclampsia or eclampsias or eclamptic or toxemia or toxemias).ti,ab. |
| 1. Gestational diabetes | "Diabetes, Gestational" [Mesh] OR "pregnancy induced diabetes" [tiab] OR “gestational diabetes” [tiab] OR “diabetes gravidarum” [tiab] | exp pregnancy diabetes mellitus/ or (pregnancy induced diabetes or gestational diabetes or diabetes gravidarum).ti,ab. |
| 1. Cardiac ultrasound | "Echocardiography "[Mesh] OR Echocardiography [tiab] | exp echocardiography/ or echocardiography.ti,ab. |
| 1. Geometry | "Ventricular Remodeling"[Mesh] OR “ventricular remodeling” [tiab] OR “cardiac remodeling” [tiab] OR “cardiac adaptation” [tiab] OR “LV geometry” [tiab] OR “left ventricular geometry” [tiab] OR “cardiac geometry” [tiab] OR “cardiac dimension” [tiab] | exp heart ventricle remodeling/ or (ventricular remodeling or cardiac remodeling or cardiac adaptation or LV geometry or left ventricular remodeling or cardiac geometry or cardiac dimension).ti,ab. |
| 1. Systolic function | “Stroke volume” [Mesh] OR "Ventricular Function, Left "[Mesh] OR “Ventricular Ejection Fraction” [tiab] OR “Left ventricular function” [tiab] OR “systolic function” [tiab] OR “Ejection fraction” [tiab] | exp heart ejection fraction/ or exp heart ventricle function/ or (Ventricular Ejection Fraction or Left ventricular function or systolic function or Ejection fraction).ti,ab. |
| 1. Diastolic function | "Diastole"[Mesh] OR "Atrial Remodeling "[Mesh] OR “diastolic function” [tiab] OR “atrial remodeling” [tiab] | exp heart left ventricle filling/ or exp diastole/ or exp heart atrium remodeling/ or (diastolic function or atrial remodeling).ti,ab. |
| 1. Aorta | "Aortic Valve" [Mesh] OR "aortic valve" [tiab] OR "aortic heart valve" [tiab] | exp aortic valve/ or (aortic valve or aortic heart valve).ti,ab. |
| **Conducted search:** (1 OR 2 OR 3 OR 4 OR 5) AND (6 OR 7 OR 8 OR 9 OR 10) | | |
|  |  |  |

| **Supplemental Table 2.** Characteristics of the study population in the reference (Ref) and normotensive pregnancy (Preg) groups | | | | | | | | | | | | | | | | |
| --- | --- | --- | --- | --- | --- | --- | --- | --- | --- | --- | --- | --- | --- | --- | --- | --- |
|  |  | |  | | **Mean NP** | |  | | **Parity / *gravidity* (n)** | | | | | |  |  |
|  | **Subjects (n)** | | **Mean age (yrs)** | | **Weight (kg)** | | **Mean height (cm)** | | *Nulli-* | | *Primi-* | | *Multi-* | | **Reference** | **GA (weeks)^#^** |
| **Study** | Ref | Preg | Ref | Preg | Ref | Preg | Ref | Preg | Ref | Preg | Ref | Preg | Ref | Preg |  |  |
| Paudel *et al.* 2020 [34] | 35 | 35 | 28,8 | 28,8 | - | - | 161 | 161 | - | - | - | - | - | - | PP (24 weeks) | 34 |
| Tasar *et al.* 2019 [35] | 47 | 47 | 29 | 29 | - | - | - | - | 47 | 47 | - | - | - | - | PP (6 months) | 1^st^-3^rd^ trim |
| Meah *et al.* 2019 [36] | 18 | 14 | 28 | 32 | - | - | 166 | 167 | 18 | 14 | - | - | - | - | NP controls | 22-26 |
| Iacobaeus *et al.* 2018 [37] | 52 | 52 | 32 | 32 | - | - | 167 | 167 | 52 | 52 | - | - | - | - | PP (9 months) | 1^st^-3^rd^ trim |
| Hieda *et al.* 2018 [38] | 25 | 25 | 31 | 31 | 61.2 | 61.2 | 163 | 162 | - | - | - | - | - | - | PC | 4-36 |
| Sengupta *et al.* 2017 [39] | 20 | 35 | 25 | 23 | 58 | - | - | - | 20 | - | - | 15* | - | 15* | NP controls | 1^st^-3^rd^ trim |
| Adeyeye *et al.* 2016 [40] | 100 | 100 | 28 | 28 | 64 | - | - | - | - | - | - | - | - | - | NP controls | 15-35 |
| Melchiorre *et al.* 2016 [5] | 50 | 559 | 30 | 32 | 56 | - | 167 | 165 | 50 | 559 | - | - | - | - | NP controls | 11-39 |
| Ando *et al.* 2016 [22] | 21 | 74 | 30 | 29 | - | - | - | - | - | - | - | - | - | - | NP controls | 21 & 33 |
| Cong *et al.* 2015 [25] | 30 | 43 | 31 | 29 | - | - | - | - | *-* | *-* | - | - | - | - | NP controls | 14-38 |
| Song *et al.* 2015 [27] | 50 | 50 | 29 | 29 | - | - | 163 | 163 | - | - | - | - | - | - | PP (6 months) | 36 |
| Papadopoulou *et al.* 2014 [41] | 11 | 27 | 30 | 30 | - | - | - | - | - | - | - | - | - | - | NP controls | 8-36 |
| Ducas *et al.* 2014 [42] | 34 | 34 | 29 | 29 | - | - | - | - | - | - | - | - | - | - | PP (16 weeks) | 34 |
| Tso *et al.* 2014 [21] | 24 | 92 | 30 | 27 | 73 | - | 162 | 162 | - | - | - | - | - | - | NP controls | 22 & 34 |
| Bazan *et al.* 2013 [43] | 48 | 47 | 31 | 29 | 62 | - | 162 | 155 | - | - | - | - | - | - | NP controls | 35-38 |
| Estensen *et al.* 2013 [44] | 63 | 65 | 32 | 32 | - | - | 168 | 168 | - | 38 | - | 21 | - | 6 | PP (6 months) | 14-36 |
| Dennis *et al.* 2012 [45] | 20 | 40 | 29 | 32 | - | - | - | - | 18 | 10 | - | - | 2 | 30 | NP controls | 36 |
| Yosefy *et al.* 2012 [23] | 20 | 40 | 31 | 31 | - | - | - | - | - | - | - | - | - | - | NP controls | 28 & 38 |
| Yoon *et al.* 2011 [46] | 23 | 32 | 28 | 25 | - | - | - | - | - | - | - | - | - | - | NP controls | 28 |
| Pandey *et al.* 2010 [18] | 22 | 46 | 26 | 26 | 54 | 54 | 164 | 164 | - | 26 | - | 8 | - | 12 | PP (8-12 weeks) | 15-40 |
| Hamad *et al.* 2009 [47] | 30 | 30 | 31 | 31 | 73 | 73 | 167 | 167 | 30 | 30 | - | *-* | - | - | PP (3-6 months) | 33 |
| Valensise *et al.* 2008 [48] | 1119 | 1119 | 32 | 32 | - | - | 164 | 164 | 1119 | 1119 | - | - | - | - | PP (1 year) | 24 |
| Bamfo *et al.* 2007 [10] | 19 | 104 | 30 | 30 | - | - | 164 | 167 | 16* | 62* | - | - | - | - | NP controls | 11-38 |
| Freire *et al.* 2006 [49] | 13 | 13 | 30 | 30 | - | - | - | - | - | - | - | - | - | - | PP (19 weeks) | 34 |
| Fok *et al.* 2006 [32] | 29 | 35 | 31 | 31 | - | - | - | - | - | - | *-* | *-* | - | - | PP (6-8 weeks) | 8-36 |
| Schannwell *et al.* 2003 [50] | 51 | 51 | 26 | 26 | - | - | - | - | - | - | *26* | *26* | - | - | PP (8 weeks) | 9-33 |
| Moran *et al.* 2002 [51] | 30 | 30 | 32 | 32 | - | - | 165 | 165 | 0.61† | 0.61† | - | *-* | - | - | PP (12-14 weeks) | 10-38 |
| Schannwell *et al.* 2002 [52] | 46 | 46 | 28 | 28 | - | - | - | - | - | - | *-* | *-* | - | - | PP (8 weeks) | 1^st^-3^rd^ trimester |
| Simmons *et al.* 2002 [53] | 44 | 44 | 29 | 29 | - | - | 163 | 163 | - | - | *44* | *44* | - | - | PP (13 weeks) | 1^st^-3^rd^ trimester |
| Kametas *et al.* 2001 *[54]* | 19 | 125 | 32 | 31 | - | - | 168 | 165 | 12* | 113* | *-* | *-* | - | - | NP controls | 9-42 |
| Borghi *et al.* 2000 *[55]* | 10 | 35 | 30 | 31 | 57 | - | 165 | 162 | - | - | - | 1.63† | - | - | NP controls | 31 |
| Mesa *et al.*1999 *[56]* | 8 | 36 | 32 | 32 | - | - | - | - | - | - | *-* | *-* | - | - | PP (7 weeks) | 1^st^-3^rd^ trimester |
| Sadaniantz *et al.* 1992 *[57]* | 28 | 28 | 32 | 31 | - | - | 164 | 164 | - | - | *-* | *-* | - | - | PP (9 weeks) | 34 |
| Escudero  *et al.* 1988 [58] | 10 | 10 | 21 | 27 | - | - | - | - | 10 | 10 | *-* | *-* | - | - | NP controls | 26-42 |
| *Only the first author is given for each study. Parity is presented in normal font; gravidity is presented in italic font. †Mean value. NP, non-pregnant. PP, postpartum. GA, gestational age. *Parity only reported for those women that completed the follow-up or parity only known for a subgroup of the study population. #, reported in weeks unless stated otherwise.* | | | | | | | | | | | | | | | | |

**Supplemental Table 3.** Quality assessment of the included studies

| **Domain** | **Items for consideration** | Paudel *et al,* 2020[34] | Meah *et al.* 2019[36] | Tasar *et al.* 2019[35] | Hieda *et al.* 2018[38] | Iacobaeus *et al*. 2018 [37] | Sengupta *et al.* 2017 [39] | Adeyeye *et al.* 2016 [40] | Melchiorre *et al.* 2016 [5] | Ando  *et al.* 2015 [22] | Cong *et al.* 2015 [25] |
| --- | --- | --- | --- | --- | --- | --- | --- | --- | --- | --- | --- |
| **Study Participation** | Adequate description of parity or gravidity | - | + | + | - | + | + | - | + | - | - |
|  | Adequate description of health or comorbidities of participants | + | + | + | + | + | + | + | + | + | + |
|  | Clear reporting of weeks amenorrhea at measurements | + | + | + | + | + | + | + | + | - | + |
|  | Adequate description of ethnicity in the study population | - | + | - | + | - | - | - | + | - | - |
|  | Height of the study participants reported | + | + | - | + | + | - | - | + | - | - |
|  | Non-pregnant weight/BMI reported of the study participants | - | - | - | + | - | - | - | - | - | - |
|  | Adequate description of medication or supplements used by the study population | + | + | + | - | - | - | - | - | - | + |
|  | Adequate description of participant recruitment | - | + | - | - | - | - | + | + | + | - |
|  | Adequate description of inclusion and exclusion criteria | + | + | + | + | + | + | + | + | + | + |
| **Study Attrition** | Reasons for loss to follow-up/drop-out are provided | ? | ? | + | - | + | + | + | ? | ? | - |
|  | Adequate description of participants lost to follow-up / differences between participants who completed the study and drop-outs | ? | ? | + | - | - | - | - | ? | ? | - |
| **Variable Measurements** | Method of measurement is adequately valid and reliable | + | + | + | + | + | + | + | + | + | + |
|  | The methods and setting are the same for all study participants and throughout follow-up | + | + | + | + | + | + | + | + | + | + |
| **Data Reporting** | Time frame of measurements are reported as mean | + | - | - | - | - | - | + | - | + | + |
| **Study Design** | Study used a longitudinal study design | + | - | + | + | + | + | + | - | - | + |
|  | Multiple (>2) longitudinal pregnant measurements during pregnancy of the variable | - | - | + | - | + | + | + | - | - | + |
|  | Reference value was a pre-pregnant measurement of the variable | - | - | - | + | - | - | - | - | - | - |
|  | **Score percentage** | 53% | 59% | 65% | 59% | 59% | 53% | 59% | 53% | 35% | 53% |
|  | **Score** | **MQ** | **MQ** | **HQ** | **MQ** | **MQ** | **MQ** | **MQ** | **MQ** | **MQ** | **MQ** |

**Supplemental Table 3 (continued).** Quality assessment of the included studies

| Song *et al.* 2015 [27] | Papadopoulou *et al.* 2014 [41] | Ducas *et al.* 2014 [42] | Tso *et al.* 2014 [21] | Bazan *et al.* 2013 [43] | Estensen *et al.* 2013 [44] | Dennis *et al.* 2012 [45] | Yosefy *et al.* 2012 [23] | Yoon *et al.* 2011 [46] | Pandey *et al.* 2010 [18] | Hamad *et al.* 2009 [47] | Valensise *et al.* 2008 [48] | Bamfo *et al.* 2007 [10] | Freire *et al.* 2006 [49] | Fok *et al.* 2006 [32] | Schannwell *et al.* 2003 [50] | Moran *et al.* 2002 [51] | Schannwell *et al.* 2002 [52] | Simmons *et al.* 2002 [53] | Kametas *et al.* 2001 [54] | Borghi *et al.* 2000 [55] | Mesa *et al.*1999 [56] | Sadaniantz *et al.* 1992 [57] | Escudero *et al.* 1988 [58] |
| --- | --- | --- | --- | --- | --- | --- | --- | --- | --- | --- | --- | --- | --- | --- | --- | --- | --- | --- | --- | --- | --- | --- | --- |
| - | - | + | - | - | + | + | - | - | + | + | + | - | - | - | + | + | - | + | + | + | - | - | + |
| + | + | + | + | + | + | + | + | + | + | + | + | - | + | + | + | + | + | - | + | + | + | - | + |
| + | + | + | + | + | + | + | + | + | + | + | + | + | + | + | + | + | + | + | + | + | + | + | + |
| + | - | - | - | - | + | + | - | - | - | - | - | - | - | - | - | - | - | - | - | - | - | - | - |
| + | - | - | + | + | + | - | - | - | + | + | + | + | - | - | - | + | - | + | + | + | - | + | - |
| - | - | + | - | - | - | - | - | - | + | - | + | - | - | - | - | - | - | - | - | - | - | - | - |
| - | - | - | - | + | + | + | - | - | - | - | - | - | + | - | - | + | - | + | - | + | - | - | - |
| - | - | + | + | - | + | + | + | + | + | + | + | - | + | + | - | + | - | + | + | + | - | + | - |
| + | + | + | + | + | + | + | + | + | + | + | + | + | + | + | + | + | + | + | - | + | - | + | - |
| + | + | - | ? | ? | + | ? | ? | ? | + | - | + | ? | - | + | - | - | - | + | ? | ? | + | - | ? |
| + | + | - | ? | ? | - | ? | ? | ? | - | - | - | ? | - | - | - | - | - | - | ? | ? | - | - | ? |
| + | + | + | + | + | + | + | + | + | + | + | + | + | + | + | + | + | + | + | + | + | + | + | + |
| + | + | + | + | + | + | + | + | + | + | + | + | + | + | + | + | + | + | + | + | + | + | + | + |
| + | - | + | + | - | - | + | + | + | - | + | + | - | + | - | + | - | + | + | + | + | + | + | - |
| + | + | + | - | - | + | - | - | - | + | + | + | - | + | + | + | + | + | + | - | - | + | + | - |
| - | + | - | - | - | + | - | - | - | - | - | - | - | - | + | + | + | + | + | - | - | + | - | - |
| - | - | - | - | - | - | - | - | - | - | - | - | - | - | - | - | - | - | - | - | - | - | - | - |
| 65% | 53% | 59% | 47% | 41% | 76% | 59% | 41% | 41% | 65% | 59% | 71% | 29% | 53% | 53% | 53% | 65% | 47% | 71% | 47% | 59% | 47% | 47% | 29% |
| **HQ** | **MQ** | **MQ** | **MQ** | **MQ** | **HQ** | **MQ** | **MQ** | **MQ** | **HQ** | **MQ** | **HQ** | **LQ** | **MQ** | **MQ** | **MQ** | **HQ** | **MQ** | **HQ** | **MQ** | **MQ** | **MQ** | **MQ** | **LQ** |

**Supplemental Figure 1.** Forest plot of E-peak change during normotensive pregnancy

**Supplemental Figure 2.** Forest plot of absolute E-peak values in normotensive pregnancies

**Supplemental Figure 3.** Forest plot of A-peak change during normotensive pregnancy

**Supplemental Figure 4.** Forest plot of absolute A-peak values during normotensive pregnancy

**

**

**Supplemental Figure 5.** Forest plot of left atrial volume change during normotensive pregnancy

**Supplemental Figure 6.** Forest plot of absolute left atrial volumes during normotensive pregnancy

**Supplemental Figure 7.** Forest plot of E/e’ ratio change during normotensive pregnancy

**Supplemental Figure 8.** Forest plot of absolute E/e’ ratio during normotensive pregnancy
